# P2X7 Activation Enhances Lipid Accumulation During Adipocytes Differentiation Through Suppressing the Expression of Sirtuin-3, Sirtuin-5, and Browning Genes

**DOI:** 10.3389/fphar.2022.852858

**Published:** 2022-04-06

**Authors:** Chien-Hsieh Chiang, Ching-Yuan Cheng, Yi-Ting Lien, Kuo-Chin Huang, Wan-Wan Lin

**Affiliations:** ^1^ Graduate Institute of Pharmacology, National Taiwan University College of Medicine, Taipei, Taiwan; ^2^ Department of Family Medicine, National Taiwan University Hospital & College of Medicine, Taipei, Taiwan; ^3^ Graduate Institute of Medical Sciences, Taipei Medical University, Taipei, Taiwan

**Keywords:** adipocyte differentiation, adipogenesis, browning, lipid accumulation, P2X7, sirtuins

## Abstract

P2X7 signaling has been explored in adipose tissue because of its potential to promote ATP-activated inflammatory cascades during obesogenic environments. However, limited literature has investigated the role of the P2X7 receptor in lipid metabolism during adipocyte differentiation. This study sought to explore the regulatory roles of P2X7 in adipocytes. This study utilized the *in vitro* 3T3-L1 differentiation model. Lipid accumulation, intracellular triglyceride, and extracellular glycerol were determined. The selective P2X7 agonist BzATP and antagonist A438079 were administered to investigate the functions of P2X7. We found that the expression of P2X7 and the lipid accumulation increased during adipocyte differentiation from D0 to D4. When administered at D0/D2, A438079 attenuated, while BzATP enhanced the degree of lipid accumulation during adipocyte differentiation. Neither did BzATP and A438079 administration affect the expression of PPARγ and C/EBPα genes that increased at D4. In addition, both intracellular triglyceride and extracellular glycerol levels at D4 were reduced by A438079 treatment and enhanced by BzATP administration. When administered at stage 2 of adipocyte differentiation, BzATP consistently enhanced lipid accumulation and intracellular triglyceride and extracellular glycerol levels without affecting mRNA and protein levels of PPARγ and C/EBPα that increased at D4. However, treating A438079 or BzATP at D4 did not affect intracellular triglyceride formation and extracellular glycerol release in differentiated adipocytes at D7. Notably, BzATP administration at stage 2 exerted a concentration-dependent inhibition on the enhanced expression of PRDM16, PGC-1α, and UCP-1 at D4. Furthermore, BzATP administration at D0/D2 inhibited the protein and mRNA levels of sirtuin-3/5 at D4. BzATP treatment at stage 2 also suppressed the mRNA levels of sirtuin-3/5 genes upregulated by insulin. In conclusion, this study demonstrated P2X7 enhances lipid accumulation during adipogenesis by suppressing the expression of sirtuin-3/5 and the browning genes.

## Introduction

Adipose tissue expansion leads to obesity through differentiation from fibroblast-like preadipocytes (adipogenesis) or hypertrophy of existing adipocytes (Ghaben and Scherer, 2019; Vishvanath and Gupta, 2019). Adipocyte differentiation involves a temporally regulated set of gene-expression events. Among them, peroxisome proliferator-activated receptor *γ* (PPARγ) and members of CCAAT enhancer-binding proteins (C/EBPs) functionally synergize in adipogenesis. PPARγ is the principal regulator of adipogenesis, and C/EBPs function at least in part by modulating PPARγ expression (Mota de Sá et al., 2017; Ghaben and Scherer, 2019). In addition, PPARγ is required to maintain the differentiated state (Imai et al., 2004; Lefterova et al., 2014). Expression of a dominant-negative PPARγ in mature 3T3-L1 adipocytes causes de-differentiation with loss of lipid accumulation and decreased expression of adipocyte markers like insulin-dependent glucose transporter (GLUT4), insulin receptor substrate (IRS), and C/EBPα. The activation of the transcription factor C/EBPα is an essential downstream effect of PPARγ (Ghaben and Scherer, 2019). Furthermore, C/EBPβ and C/EBPδ play early catalytic roles in the differentiation cascade that induces the activation of the gene encoding C/EBPα (Yeh et al., 1995; Wang and Hai, 2015). These transcription factors can bind to several adipocyte gene promoters during differentiation, including PPARγ (Ntambi and Young-Cheul, 2000; Wang and Hai, 2015).

White adipocytes act as storage cells upon excess calories. In addition to adipolysis (also called lipolysis) that refers to the degradation of triglyceride (TG) to fatty acid and glycerol in white adipocytes, the browning effect of adipocytes is another crucial process in response to positive energy balance (Verkerke and Kajimura, 2021). The browning of white adipose tissue (WAT) leads to reduced lipid accumulation and increased production of brown fat-like adipocytes within WAT, called beige adipocytes (Bartelt and Heeren, 2014; Abdullahi and Jeschke, 2016). These beige adipocytes have a brown adipose tissue (BAT)-like phenotype and non-shivering thermogenic actions, with PR domain containing 16 (PRDM16), uncoupling protein 1 (UCP1), PPAR-γ coactivator (PGC) -1α, and silent information regulators (SIRTs) highly expressed (Bartelt and Heeren, 2014; Demine et al., 2019; Shuai et al., 2019). Among them, mitochondrial sirtuin-3 (SIRT3) and sirtuin-5 (SIRT5) counteract oxidative stress and exert interconnected roles in metabolic networks (Ji et al., 2021). Both SIRT3 and SIRT5 promote adaptive thermogenesis in BAT (Favero et al., 2018; Sebaa et al., 2019; Shuai et al., 2019), although the exact mechanisms are still unclear. The inherent property of dissipating energy in brown and beige adipocytes might be beneficial in counteracting obesity and other metabolic diseases.

The purinergic receptors regulate cellular functions through their capacity to use extracellular ATP, adenosine, and other nucleotides and nucleosides for triggering intracellular signal transduction mechanisms (Coccurello and Volonté, 2020). Among all purinergic receptors, the P2X7 receptor is a ligand-gated ion channel activated by high extracellular ATP concentration (Burnstock, 2006). Because ATP is constitutively and passively released upon tissue injury or cell damage, the ATP-mediated signaling downstream from activation of P2X7 (possessing a higher dissociation constant for ATP compared with other P2 receptors) is related to the damage-associated molecular patterns. Activation of P2X7 for milliseconds rapidly triggers K^+^ and Ca^2+^ ions across the plasma membrane (Seeland et al., 2015), leading to membrane permeabilization and a large, non-selective pore formation. Such a large pore allows molecules with molecular weight up to 900 Da to enter the cells. Consequently, it initiates several cellular events, including nucleotide-binding domain leucine-rich repeat family pyrin domain-containing protein 3 (NLRP3) inflammasome formation, the opening of pannexin 1 and connexin hemichannels, membrane blebbing, reactive oxygen species (ROS) production, loss of mitochondrial membrane potential, and eventually cellular death (Schroder and Tschopp, 2010; Idzko et al., 2014; Lin et al., 2015; Di Virgilio et al., 2017; Sekar et al., 2018; Chiu et al., 2022). P2X7 is widely expressed in several tissues, but it is not highly expressed in white or brown adipose tissues unless activated upon pharmacological agents or pathological environments (Jain and Jacobson, 2021). Considering that P2X7 is unique in activating inflammatory cascades and chronic inflammation contributes to obesity development (Xu et al., 2003), it is interesting to explore the relevance of P2X7 in adipose tissues. A human study revealed that the expression of P2X7 in visceral and subcutaneous adipose tissue of patients with metabolic syndrome was higher than controls (Madec et al., 2011). Consistently, both mRNA and protein levels of *P2X7* in WATs were significantly increased in *ob*/*ob* mice relative to age-matched WT lean mice (Sun et al., 2012). The *P2X7* mRNA level was elevated starting at six weeks of 60% high-fat diet (HFD), and the P2X7 protein was induced in adipose tissue after eight weeks of HFD (Sun et al., 2012). Furthermore, P2X7-deficient mice on a regular diet have increased adipose tissue mass but decreased energy expenditure and fat oxidation (Beaucage et al., 2014; Giacovazzo et al., 2018). However, P2X7-deficient mice on HFD exhibited no changes in metabolic phenotypes, inflammatory responses, or inflammasome activation compared with the wild-type controls (Sun et al., 2012). On the other hand, a study focused on the role of P2X7 in the development of non-alcoholic fatty liver disease showed that P2X7 gene deletion protected mice from HFD-induced non-alcoholic steatohepatitis, possibly through blunted activation of NLRP3 inflammasome (Blasetti Fantauzzi et al., 2017). Recent studies also suggest that P2X7 may regulate lipid metabolism in microglia or adipocytes through modulating ROS production (Bartlett et al., 2013), mitochondrial activity (Seeland et al., 2015), and autophagy (Fabbrizio et al., 2017). Nonetheless, little literature has directly characterized the nature of P2X7 regarding adipogenesis and lipid metabolism. Therefore, this study utilized mouse 3T3-L1 adipocytes as the cell model to decipher the roles of P2X7 in adipocyte differentiation and lipid metabolism.

## Materials and Methods

### Reagents and Antibodies

DMEM, fetal bovine serum (FBS), bovine serum (BS), and trypsin-EDTA were purchased from ThermoFisher Scientific (United States). Penicillin (10000 units/ml)- streptomycin (10 mg/ml) solution was purchased from Biological Industries (Israel). Nigericin, benzoylbenzoyl-ATP (BzATP), and A438079 were purchased from Sigma-Aldrich (United States). Primary antibodies against β-actin, P2X7, PPARγ, C/EBPα, PRDM16, UCP1, PGC-1α, and horseradish peroxidase-conjugated anti-goat IgG secondary antibody were purchased from Santa Cruz Biotechnology, Inc. (United States). Primary antibodies against SIRT3, SIRT5, phospho-insulin receptor (IR), IR, phospho-insulin receptor substrate 1 (IRS-1), IRS-1, phospho-Akt, Akt, and peroxidase-conjugated anti-rabbit/mouse IgG secondary antibody were purchased from Cell Signaling Technology, Inc (United States).

### Cell Model

The mouse 3T3-L1 cell culture line cloned from mouse embryo fibroblasts is a well-known model to study adipogenesis and lipogenesis (Student et al., 1980). 3T3-L1 preadipocytes were cultured in DMEM supplemented with 10% BS, 1 mM sodium pyruvate, and 10% penicillin-streptomycin solution. For standard induction of differentiation, 3T3-L1 preadipocytes were cultured in DMEM with 10% FBS and 1 mM sodium pyruvate and kept in 100% confluent stage for two days before starting the differentiation process by treating cells with a defined adipogenic cocktail MDI (Student et al., 1980; Ntambi and Young-Cheul, 2000). On D0, cells were induced to differentiate using 0.5 mM 3-isobutyl-1-methylxanthine (IBMX), 5 μM dexamethasone (DEX), and 10 μg/ml insulin. At the end of D2, DEX and IBMX were removed, and cells were cultured in a 1 μg/ml insulin-containing culture medium for two days. The medium was changed with DMEM every two days until the cells were collected for experimental use.

### Oil Red O Staining

Differentiated adipocytes at D4 were washed with phosphate-buffered saline (PBS), fixation with 10% formalin for 1 h at room temperature, and rewashed three times with deionized water. A mixture of Oil Red O solution (0.6% Oil Red O dye in isopropanol) and water in a 6:4 ratio was layered onto cells for 20 min, followed by washing four times with deionized water, and images were captured under a microscope. The dye was dissolved by isopropanol, and the OD values at 510 nm were measured using a microplate reader.

### Quantitative Real-Time Polymerase Chain Reaction

Total RNA was isolated from cells at D0, the end of D2 or D4 using a total RNA extraction reagent (REzol™ C&T, Protech Technology Enterprise Co., Ltd.). RNA (1 μg) was converted to cDNA using Moloney Murine Leukemia Virus Reverse Transcriptase (M-MLV RT, Promega Corporation, United States). FastStart universal SYBR^®^ Green Master (Roche Diagnostics GmbH, Germany) was employed to quantitatively determine transcription levels of genes with qRT-PCR (QuantStudio™ 5, Applied Biosystems™). PCR reactions were run in duplicate for each sample, and transcription levels of all genes were normalized to the level of β-actin. The change of mRNA expression was calculated by using the 2^−ΔΔCt^ method. Sequences of primer sets used in this study are listed in Table 1.

**TABLE 1:** Sequences of primer sets.

Name	Sequences (5′ to 3′)
P2X7	F: AGC​ACG​AAT​TAT​GGC​ACC​GT
	R: CCC​CAC​CCT​CTG​TGA​CAT​TCT
PPARγ2	F: TCG​CTG​ATG​CAC​TGC​CTA​TG
	R: GAG​AGG​TCC​ACA​GAG​CTG​ATT
C/EBPα	F: CCGGGAGAACTCTAACTC
	R: GATGTAGGCGCTGATGT
C/EBPβ	F: GCAAGAGCCGCGACAAG
	R: GGCTCGGGCAGCTGCTT
PRDM16	F: GAT​GGG​AGA​TGC​TGA​CGG​AT
	R: TGA​TCT​GAC​ACA​TGG​CGA​GG
PGC-1α	F: AGC​CGT​GAC​CAC​TGA​CAA​CGA​G
	R: GCT​GCA​TGG​TTC​TGA​GTG​CTA​AG
UCP1	F: CCT​GCC​TCT​CTC​GGA​AAC​AA
	R: GTA​GCG​GGG​TTT​GAT​CCC​AT
SIRT3	F: GCT​GCT​TCT​GCG​GCT​CTA​TAC
	R: GAA​GGA​CCT​TCG​ACA​GAC​CGT
SIRT5	F: GTC​TCC​TGT​GGG​ATT​CCT​GA
	R: ACA​CAG​AGA​CGG​CTG​GAA​CT
β-actin	F: CGG​GGA​CCT​GAC​TGA​CTA​CC
	R: AGG​AAG​GCT​GGA​AGA​GTG​C

PPARγ, peroxisome proliferator-activated receptor γ; C/EBP, CCAAT enhancer-binding proteins; PRDM16, PR/SET Domain 16; PGC-1α, PPARγ, coactivator 1α; UCP1, uncoupling protein 1; SIRT, silent information regulators.

### Immunoblot Analysis

Cell lysates were prepared using RIPA buffer (50 mM Tris at pH 7.6, 150 mM NaCl, 0.1% SDS, 0.1% NaDOc, 1% Triton X-100, 2 mM NaF, 2 mM EDTA, 2 mM Na_3_VO_4_, and 1 mM PMSF) by homogenization and centrifugation at 13,000 r.p.m. for 20 min. Cell extract was diluted in 5X sample buffer (50 mM Tris at pH 6.8, 5% SDS, 37.5% glycerol, 36 mM β-mercaptoethanol, and 0.25% bromophenol blue) and heated for 10 min at 95°C before 8, 10, or 12% SDS-polyacrylamide gel electrophoresis (PAGE). After electrophoresis, samples were transferred to a polyvinylidene difluoride membrane (PVDF, GE Healthcare Life Sciences, United States) and then blocked for 1 h with TBS-T (20 mM Tris–HCl pH 7.4, 150 mM NaCl, and 0.1% Tween 20) containing 5% skim milk. The membrane was rinsed three times consecutively with TBS-T buffer, followed by incubation with 1:1000 dilutions of primary polyclonal antibodies in TBS-T buffer containing 1% skim milk. After three washes, the membrane was incubated for 1 h with horseradish peroxidase-conjugated anti-goat IgG (1:10000) or anti-rabbit/mouse IgG secondary antibody (1:10000) in TBS-T buffer containing 1% skim milk. Development was carried out using an enhanced chemiluminescence solution (Western Lightning Plus-ECL, PerkinElmer, Inc.). The band intensities were quantified using Image Lab software (Bio-Rad).

### Flow Cytometry for Cell Viability

3T3-L1 preadipocyte cells were seeded in 3.5 cm dishes (1×10^5^ cells) and treated with A438079 or BzATP for 24 and 48 h, respectively. Cells were washed with PBS and then collected by 0.25% trypsinization. Cells were resuspended in Annexin V binding buffer (Biolegend, United States) and stained with 5 μL Annexin V-FITC and 10 μL propidium iodide (PI) at 37°C for 25 min. After two washes by PBS, cells were resuspended in 500 μL Annexin V binding buffer, and the cell death was recorded using FACScalibur (BD biosciences).

### TG Quantification and Glycerol Assay

Intracellular TG content was determined by a quantification assay kit (ab65336, abcam^®^, UK). Briefly, cells were resuspended in 5% NP-40/ddH_2_O solution and heated at 90-100°C to solubilize TG. The cultured medium was collected to analyze the extracellular glycerol content using Free Glycerol Assay kit (ab65337, abcam^®^, UK). Determinations of intracellular TG and extracellular glycerol contents were carried out according to the protocols provided by the manufacturer.

### Statistical Analysis

All data were expressed as the mean ± SEM of at least three independent experiments. Statistical significance levels were determined by two-tailed Student’s t-tests (*p* < 0.05). SAS software version 9.4 (SAS Institute Inc., Cary, NC, United States) was utilized for statistical analyses.

## Results

### P2X7 Upregulation During Adipocyte Differentiation is Involved in Lipid Accumulation

**FIGURE 1:**
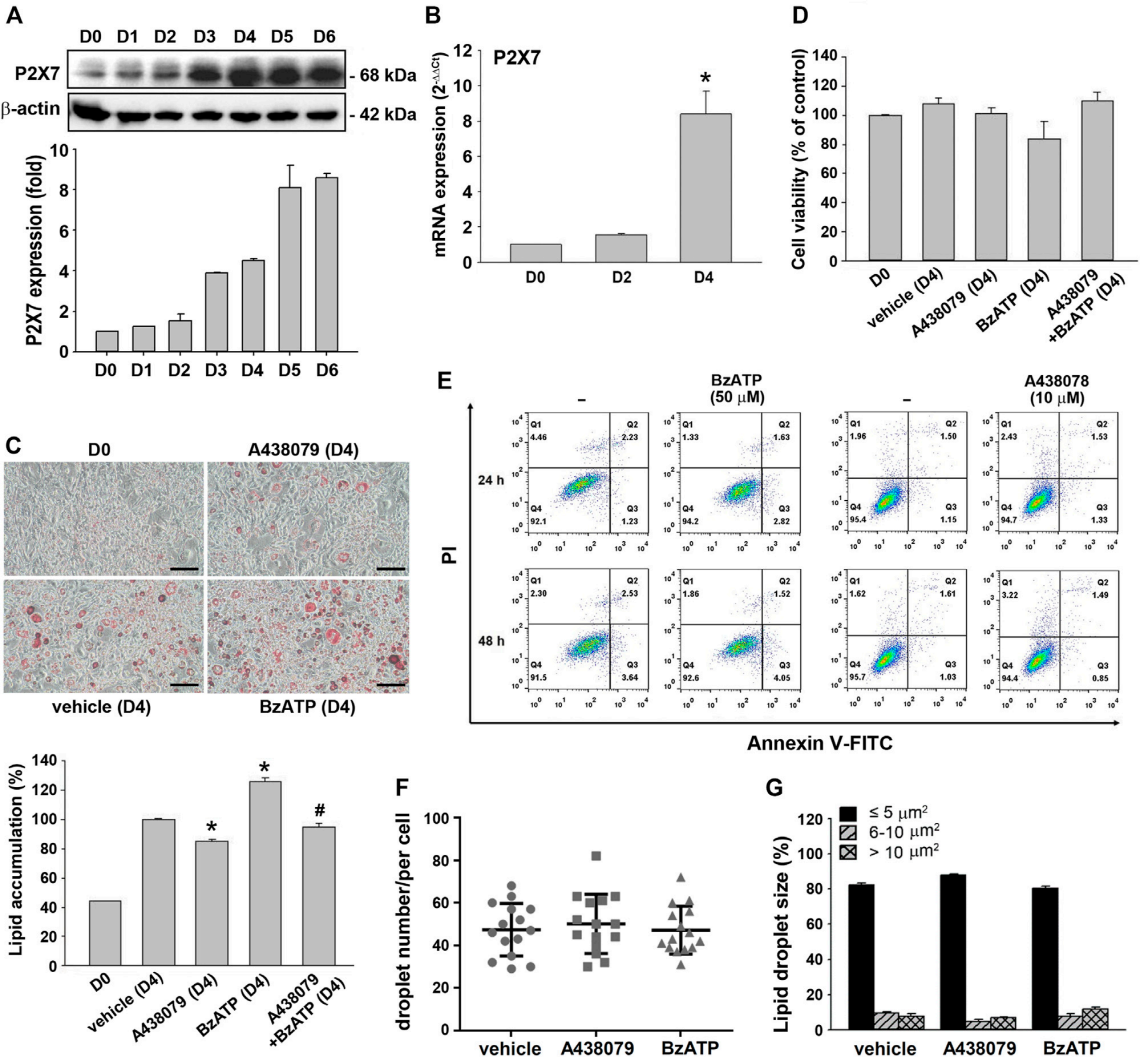
P2X7 expression and lipid accumulation during adipocyte differentiation. **(A)** The protein levels of P2X7 during adipocyte differentiation (D0-D6) were measured by western blot analysis with quantification to β-actin. **(B)** The mRNA levels of *P2X7* during adipocyte differentiation (D0, D2, and D4) were measured by qPCR. *p < 0.05 *vs*. preadipocytes (D0). **(C)** Cells were treated with A438079 (10 μM) or BzATP (50 μM) at D0 and again at the end of D2 upon the renewal of the differentiation media. After two-stage adipocyte differentiation, the intracellular lipid was stained by Oil Red O. Scale bar: 100 μm. The Oil Red O stain was extracted in isopropanol and was used to measure the value at 510 nm *p < 0.05 *vs*. vehicle-treated adipocytes (D4) and ^#^ *p* < 0.05 *vs*. adipocytes treated with BzATP (D4). **(D)** Cell viability of adipocytes at D4 was measured by Trypan Blue cell counting. **(E)** 3T3-L1 preadipocytes were treated with A438079 or BzATP for 24-48 h. Cell viability was determined by flow cytometry. The **(F)** droplet numbers and **(G)** droplet sizes in vehicle-, A438079-, and BzATP-treated cells were measured at D4 using SGcapture software (Optical Gaging Products). All quantitative data are presented as mean ± SEM from at least three independent experiments.

Using the *in vitro* 3T3-L1 preadipocyte differentiation model, we found that the P2X7 protein level increased in a time-dependent manner during adipocyte differentiation for four days. The P2X7 protein level was maintained in differentiated adipocytes from D4 to D6 (Figure 1A). Consistently, the P2X7 mRNA level significantly increased at the second stage of differentiation (from the end of D2 to the end of D4) (Figure 1B). To understand the role of P2X7 in adipocyte differentiation, we determined the effects of A438079 and BzATP, which are the selective P2X7 antagonist and agonist, respectively. First, we measured the lipid content by oil-red staining along with cell differentiation. As shown in Figure 1C, we found opposite effects of A438079 (10 μM) and BzATP (50 μM) on lipid accumulation, which was increased from preadipocytes to differentiated adipocytes. When administered at D0/D2, A438079 attenuated, while BzATP enhanced lipid accumulation during adipocyte differentiation. In the presence of A438079, the increasing effect of BzATP was blocked, suggesting the role of P2X7 in lipid accumulation upon adipocyte differentiation. In addition, we ruled out the changes in lipid accumulation resulting from cell viability. Both P2X7 agents did not alter the viable cell amount at D4 revealed by trypan blue staining (Figure 1D). The data of Annexin V/PI staining also showed no effects of BzATP (50 μM) and A438079 (10 μM) on cell viability after treatment for 24 or 48 h in 3T3-L1 preadipocytes (Figure 1E). Moreover, BzATP and A438079 administration at D0/D2 did not alter the distribution of lipid droplet numbers and sizes after four-day differentiation (Figures 1F,1G **)**.

### P2X7 Involvement in Lipid Metabolism is Not Through Regulating the Expression of PPARγ and C/EBPα

**FIGURE 2:**
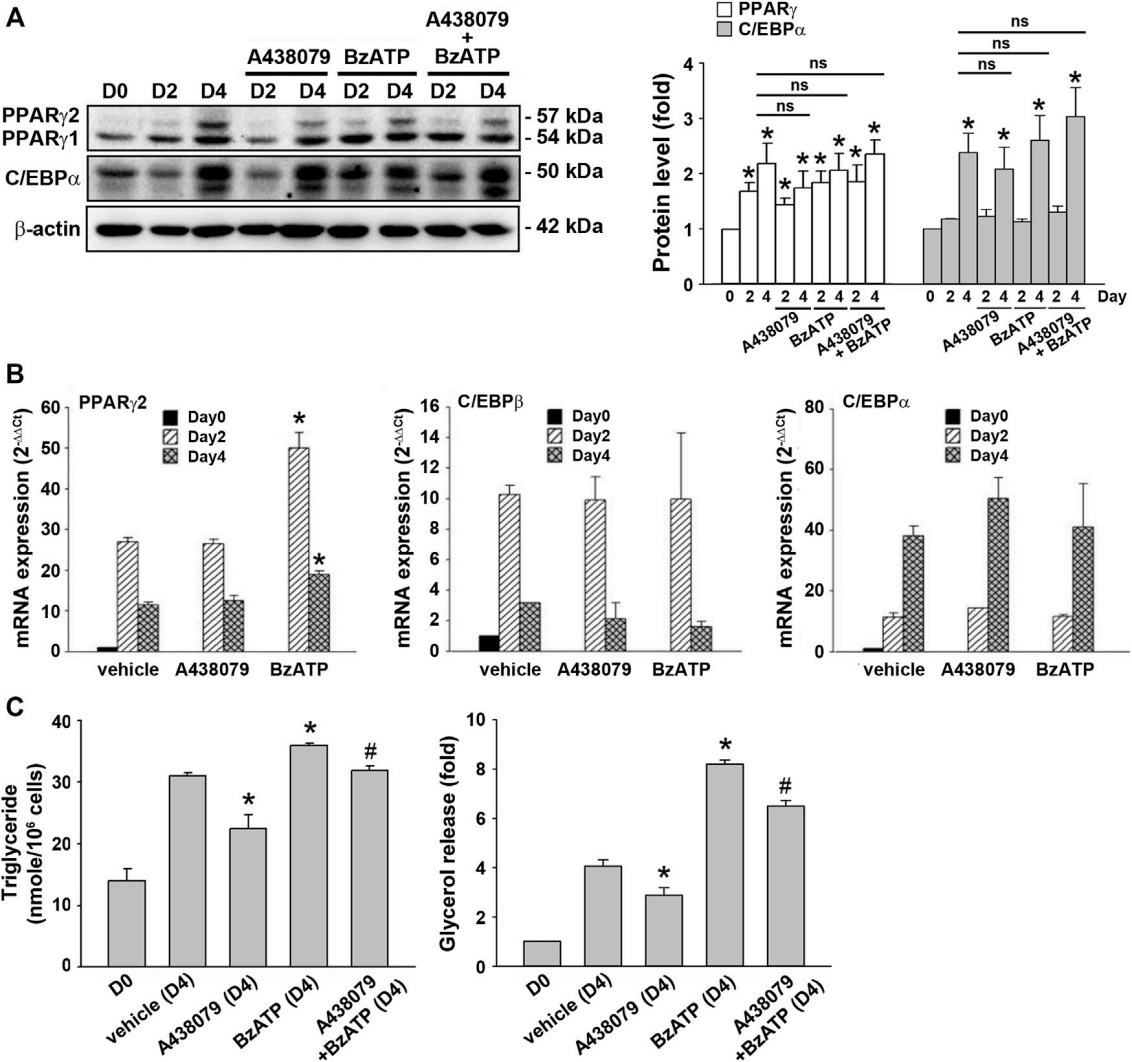
P2X7 does not affect adipocyte differentiation but enhances lipid metabolism. 3T3-L1 preadipocytes were differentiated in the two-stage model for four days. Vehicle, A438079 (10 μM) or BzATP (50 μM) were administered at D0 and again at the end of D2 upon the renewal of the differentiation media. **(A)** The protein levels of PPARγ and C/EBPα at D0, the end of D2, and the end of D4 during adipocyte differentiation were determined by western blot analysis and quantified by normalizing to β-actin from three independent experiments. *p < 0.05 *vs*. vehicle-treated preadipocytes at D0. ns: non-significant. **(B)** The gene expression of *PPARγ2*, *C/EBPβ*, and *C/EBPα* during adipocyte differentiation was measured by qPCR. *p < 0.05 *vs*. vehicle-treated adipocytes. **(C)** Levels of intracellular triglyceride and released glycerol in culture medium at the end of D4 were determined by triglyceride assay kit (Abcam) and adipolysis assay kit (Millipore), respectively. *p < 0.05 *vs*. vehicle-treated adipocytes at D4 and ^#^ *p* < 0.05 *vs*. BzATP-treated adipocytes at D4. All quantitative data are presented as mean ± SEM from at least three independent experiments.

Because lipid accumulation in this cell model depends on adipocyte differentiation and lipid metabolism balanced by lipid synthesis and lipolysis, we determined the effects of P2X7 on both events. To verify adipocyte differentiation, two sequential adipogenic factors PPARγ and C/EBPα were measured. The immunoblotting data validated the *in vitro* adipocyte differentiation in the 3T3-L1 cell model by showing marked increases in PPARγ expression from the end of D2 and C/EBPα expression at the end of D4 (Figure 2A). Compared with a maximal induction of *PPARγ* and *C/EBPβ* expression at D2 followed by a decline at D4, a continuous increase in mRNA level up to D4 was observed for *C/EBPα*. The early rise of *PPARγ* and *c/EBPβ* gene expression at D2 accounts for the sequentially remarkable gene expression of *C/EBPα* observed at D4 (Figure 2B). We found that A438079 (10 μM) and BzATP (50 μM) administration at D0/D2 did not affect the protein levels of both transcription factors during adipocyte differentiation (Figure 2A). Moreover, A438079 did not alter the upregulated mRNA levels of these transcription factors upon adipocyte differentiation since D2. BzATP increased the mRNA level of *PPARγ* at D2 and D4 but did not alter the mRNA levels of *C/EBPα* and *C/EBPβ* at D2 and D4 (Figure 2B). These data suggest that P2X7 does not affect adipocyte differentiation.

TG is the major lipid component in adipocytes. It can be metabolized to free fatty acid and glycerol by hormone-sensitive lipase. Because glycerol can be secreted *via* AQPad (aquaporin adipose), we determined intracellular TG and extracellular glycerol. As shown in Figure 2C, intracellular TG level was increased in differentiated adipocytes at D4 compared to preadipocytes. A438079 treatment attenuated, while BzATP administration increased this effect. Similarly, secreted glycerol level was higher in differentiated adipocytes at D4 than in preadipocytes, and this increase was reduced by A438079 treatment while further enhanced by BzATP administration. These findings suggest that P2X7 is involved in lipid metabolism by increasing lipid accumulation in differentiated adipocytes at D4.

### P2X7 Activation at Stage 2 of Adipocyte Differentiation is Sufficient to Enhance Lipid Metabolism Without Affecting Adipocyte Differentiation

**FIGURE 3:**
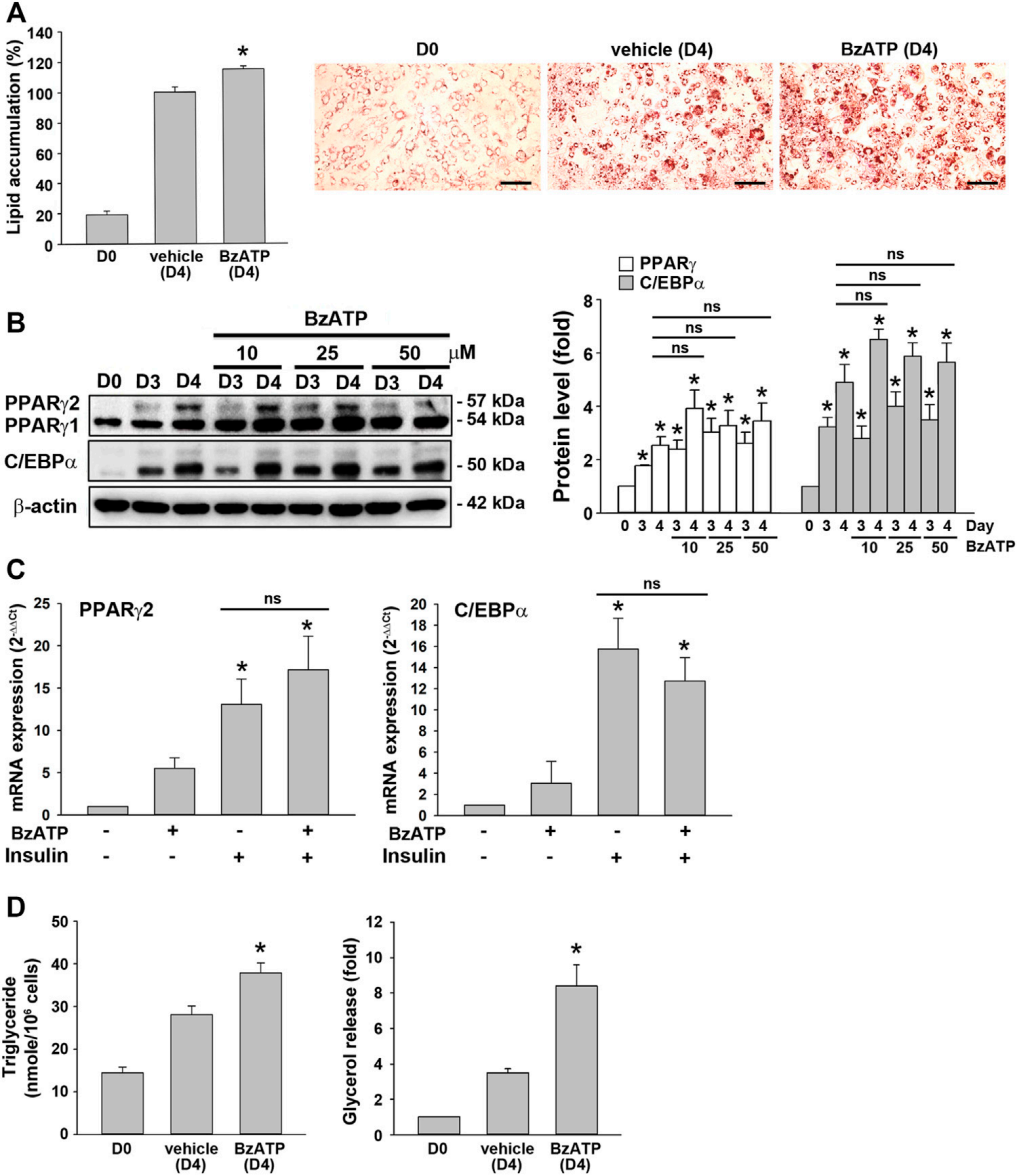
P2X7 activation at the second stage of adipocyte differentiation is sufficient to increase lipid accumulation without affecting adipogenic genes. **(A)** In the two-stage differentiation model, cells were treated with vehicle or BzATP (50 μM) at the end of D2. After 48 h (at the end of D4), cells were stained with Oil Red O to determine intracellular lipid accumulation. *p < 0.05 *vs*. vehicle-treated adipocytes (D4). Scale bar: 100 μm. **(B)** Cells after two-day first-stage differentiation were treated with BzATP (10, 25, 50 μM) for 24 h (determined at the end of D3) and 48 h (determined at the end of D4). The protein levels of PPARγ and C/EBPα were determined by western blot analysis and quantified by normalizing to β-actin from three independent experiments. *p < 0.05 vs. vehicle-treated preadipocytes at D0. ns: non-significant. **(C)** Cells after two-day first-stage differentiation were treated with BzATP (50 μM) for 24 h in the presence or absence of insulin (1 μg/ml). The mRNA levels of *PPARγ2* and *C/EBPα* were determined by qPCR. *p < 0.05 *vs*. vehicle-treated adipocytes. ns: non-significant. **(D)** The cell lysate and cultured medium described in **(A)** were collected to determine intracellular triglyceride and extracellular glycerol content. *p < 0.05 *vs*. vehicle-treated adipocytes (D4). All quantitative data are presented as mean ± SEM from at least three independent experiments.

As shown in Figure 1A, an increased P2X7 protein level during adipocyte differentiation was apparent at D3. Thus, we studied whether P2X7 activation at stage 2 of adipocyte differentiation would increase lipid accumulation. When administered at the end of D2 (stage 2), BzATP administration enhanced lipid accumulation at D4 (Figure 3A) but did not affect protein levels of PPARγ and C/EBPα at D4 (Figure 3B). Neither did BzATP treatment at stage 2 affect mRNA levels of *PPARγ* and *C/EBPα*, both enhanced by stage 2 inducer insulin (1 μg/ml) (Figure 3C). Consistently, BzATP treatment at stage 2 increased intracellular TG and extracellular glycerol levels at D4 (Figure 3D). These findings indicate that P2X7 activation at stage 2 could enhance lipid metabolism without affecting adipocyte differentiation.

**FIGURE 4:**
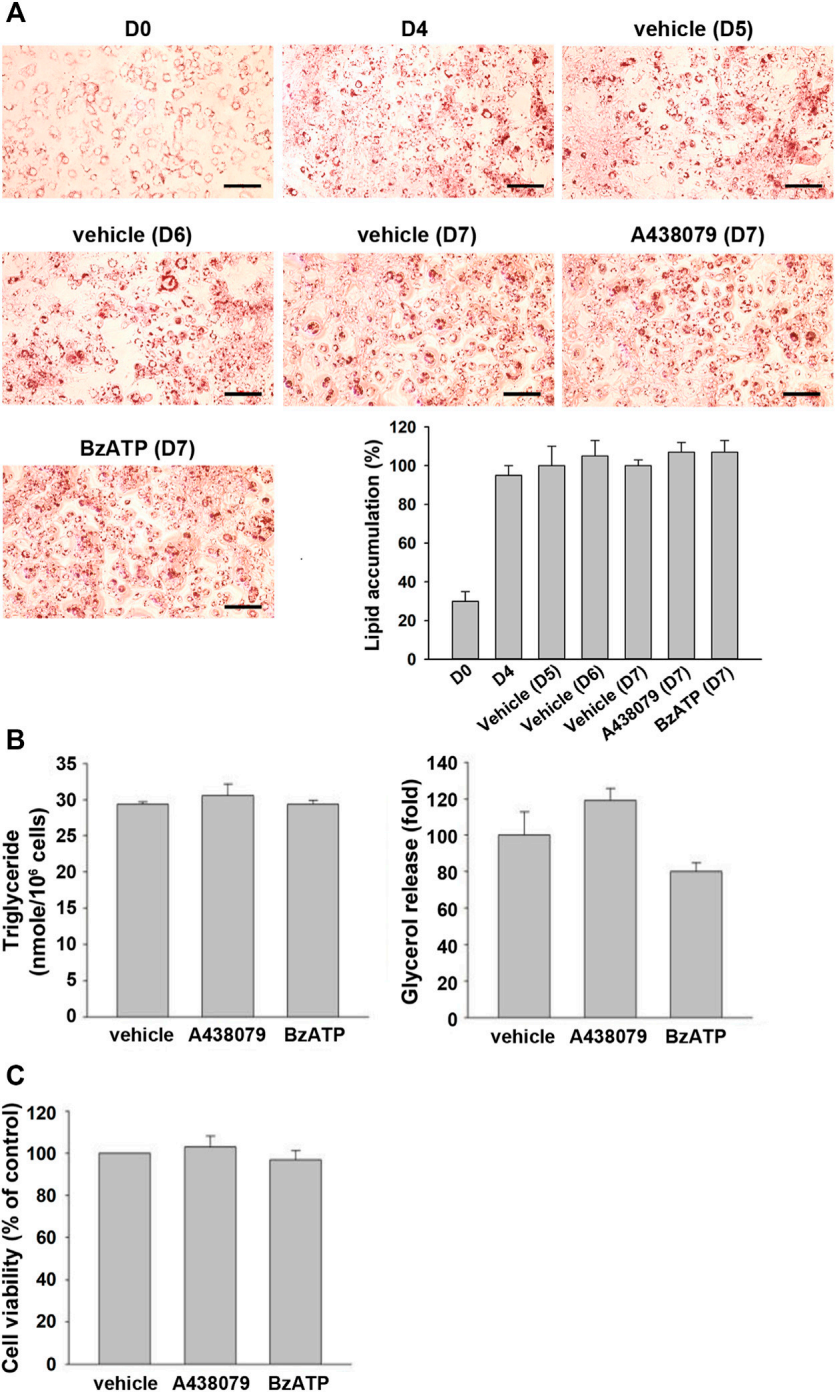
P2X7 does not affect lipid metabolism of adipocytes after four-day differentiation. After two-stage differentiation, 3T3-L1 adipocytes were cultured in a complete DMEM containing 10% FBS without insulin, but with A438079 (10 μM) or BzATP (50 μM) for additional three days. **(A)** Cells at indicated differentiation day were stained with Oil Red O and quantified to determine lipid accumulation. **(B)** Intracellular triglyceride level and glycerol release at D7 were determined. **(C)** The cell viability of differentiated adipocytes at D7 was measured by Trypan Blue cell counting. Data are presented as mean ± SEM from at least three independent experiments.

To further understand if P2X7 activation is also involved in lipid accumulation in differentiated adipocytes, we treated P2X7 agents in 3T3-L1 adipocytes having undergone the second stage of differentiation. Upon treatment of A438079 or BzATP in adipocytes at the end of D4 for three days, 3T3-L1 adipocytes still maintained similar intracellular lipid accumulation even in the absence of insulin (Figure 4A). Consistently, treating A438079 (10 μM) or BzATP (50 μM) at D4 did not affect intracellular TG formation and extracellular glycerol release in mature adipocytes at D7 (Figure 4B). The cell viability of mature adipocytes at D7 remained comparable among vehicles, BzATP, and A438079 groups (Figure 4C). These data show the homeostasis of lipid metabolism in adipocytes from D4 to D7. Overall, these findings indicate that P2X7 does not regulate lipid metabolism in differentiated adipocytes from D4 to D7, unlike its potential to promote lipid accumulation during adipocyte differentiation from D0 to D4.

### P2X7 Activation Suppresses the Browning Process During Adipocyte Differentiation

**FIGURE 5:**
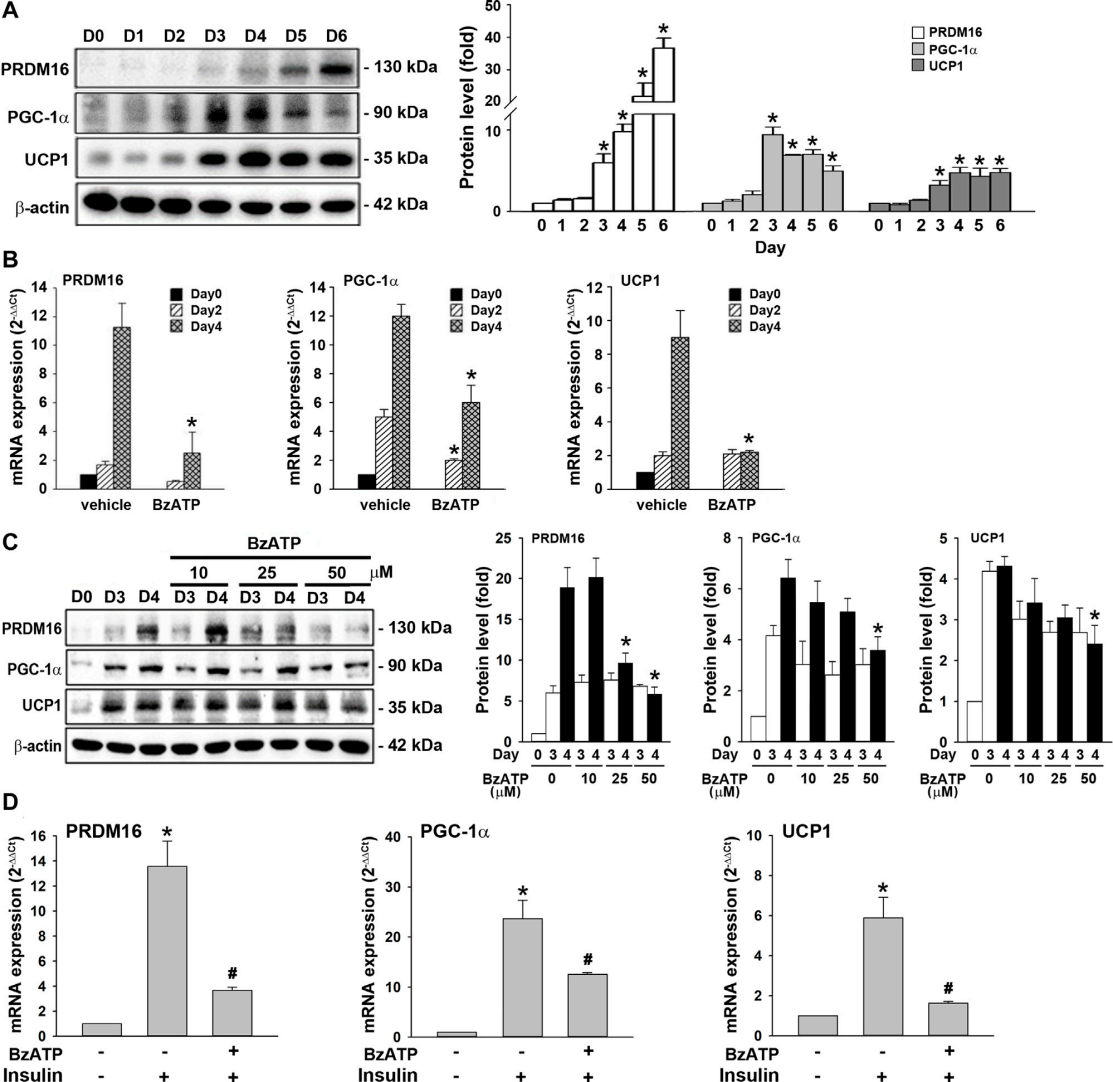
P2X7 activation downregulates the expression of PRDM16, PGC-1α, and UCP1 genes during adipocyte differentiation. **(A)** Western blot analysis of browning marker PRDM16, PGC-1α, and UCP1 during adipocyte differentiation with quantification by normalizing to β-actin. *p < 0.05 *vs*. preadipocytes at D0. **(B)** Cells were treated with BzATP (50 μM) at D0 and again at the end of D2 upon the renewal of the differentiation media. The mRNA levels of *PRDM16*, *PGC-1α*, and *UCP1* at D0, D2, and D4 were measured by qPCR. *p < 0.05 vs. vehicle-treated adipocytes at D2 or D4. **(C)** Cells after two-day differentiation (at the end of D2) were treated with insulin (1 μg/ml) with or without BzATP (10, 25, 50 μM) for 24 h (determined at the end of D3) and 48 h (determined at the end of D3). The protein levels were determined by western blot analysis with quantification by normalizing to β-actin. *p < 0.05 *vs*. vehicle-treated adipocytes at D4. **(D)** Differentiated adipocytes at the end of D2 were treated with insulin (1 μg/ml) and/or BzATP (50 μM) for 24 h. The mRNA levels of *PRDM16*, *PGC-1α*, and *UCP1* were determined by qPCR. *p < 0.05 *vs*. vehicle-treated adipocytes and ^#^ *p* < 0.05 *vs*. insulin-treated adipocytes. All quantitative data are presented as mean ± SEM from at least three independent experiments.

WAT browning reduces lipid accumulation (Bartelt and Heeren, 2014; Abdullahi and Jeschke, 2016). Given that BzATP administration enhanced lipid accumulation without directly affecting adipocyte differentiation (adipogenesis) (Figure 1C, Figure 2A), we sought to study whether P2X7 regulates the browning process. The browning process has been demonstrated during 3T3-L1 adipocyte differentiation (Kim et al., 2020). PRDM16, PGC-1α, and UCP1 are key proteins regulating this event. We observed that PRDM16, PGC-1α, and UCP1 proteins were upregulated along with adipocyte differentiation. The PRDM16 protein level kept increasing from D4 to D6, the PGC-1α protein level reached a maximum level at D3, while the UCP1 protein level reached a plateau since D4 (Figure 5A). Consistently, the mRNA levels of *PRDM16, PGC-1α*, and *UCP1* significantly increased from D2 to D4, suggesting that the browning process occurs at stage 2 (Figure 5B). We found BzATP administration at D0/D2 reduced *PRDM16, PGC-1α,* and *UCP1* mRNA levels at D4 (Figure 5B), although it did not suppress mRNA levels of adipogenic factors required for adipocyte differentiation (Figure 2B). Moreover, we detected the time- and concentration-dependent inhibition of PRDM16, PGC-1α, and UCP1 protein levels by BzATP administration at the end of D2. Meanwhile, BzATP at 50 μM exerted the most apparent effect (Figure 5C) at D4. Consistently, BzATP treatment at stage 2 of adipocyte differentiation suppressed mRNA levels of *PRDM16, PGC-1α*, and *UCP1* that were initiated by stage 2 inducer insulin (1 μg/ml) (Figure 5D). Given that downregulation of PRDM16, PGC-1α, and UCP1 by P2X7 activation leads to attenuation of the browning effect, the above findings implicate that inhibition of the browning process might be the mechanism of BzATP to increase lipid accumulation during adipogenesis.

### P2X7 Activation Downregulates SIRT3/5 Expression During Adipogenesis

**FIGURE 6:**
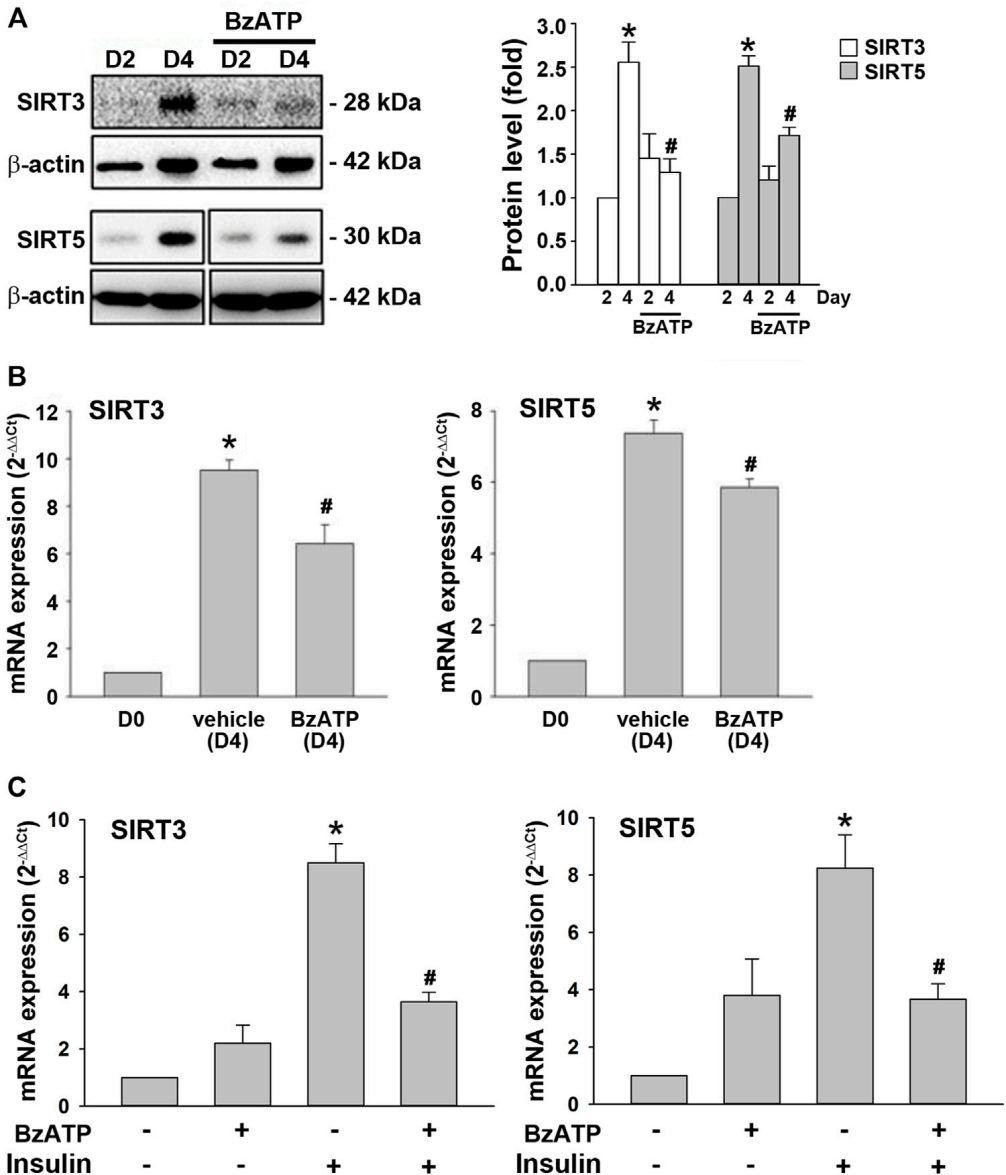
P2X7 activation downregulates the expression of *SIRT3/5* genes during adipocyte differentiation. **(A,B)** Cells were treated with BzATP (50 μM) at D0 and again at the end of D2 upon the renewal of the differentiation media. **(A)** The protein levels of SIRT3 and SIRT5 at D2 and D4 were determined by western blot analysis with quantification by normalizing to β-actin. *p < 0.05 *vs*. differentiated adipocytes at D2. ^#^ *p* < 0.05 *vs*. vehicle-treated adipocytes at D4. **(B)** The mRNA levels of *SIRT3* and *SIRT5* at D4 were determined by qPCR. *p < 0.05 *vs*. preadipocytes (D0) and ^#^ *p* < 0.05 *vs*. vehicle-treated adipocytes at D4. **(C)** Cells after two-day differentiation (at the end of D2) were treated with BzATP (50 μM) for 24 h (determined at the end of D3) in the presence or absence of insulin (1 μg/ml). The mRNA levels of *SIRT3* and *SIRT5* at D3 were determined by qPCR. *p < 0.05 *vs*. vehicle-treated adipocytes (D3) and ^#^ *p* < 0.05 *vs*. adipocytes treated with insulin (D3). All quantitative data are presented as mean ± SEM from at least three independent experiments.

Next, we were interested to understand the molecular mechanism underlying the downregulation of PRDM16 by P2X7. Sirtuins are NAD^+^-dependent deacetylases that regulate gene expression and metabolic processes (Osborne et al., 2016). Both SIRT3 and SIRT5 upregulate brown adipocyte activity or differentiation (Favero et al., 2018; Sebaa et al., 2019; Shuai et al., 2019). In adipocytes, we found that SIRT3 and SIRT5 proteins were upregulated at D4, which was suppressed by BzATP administration at D0/D2 (Figure 6A). Consistently, BzATP administration reduced the increased mRNA levels of *SIRT3* and *SIRT5* at D4 during adipogenesis (Figure 6B). Moreover, BzATP treatment at stage 2 of adipocyte differentiation suppressed the mRNA levels of *SIRT3* and *SIRT5* that were upregulated by stage 2 inducer insulin (1 μg/ml) (Figure 6C). Collectively, these findings indicate that P2X7-mediated enhancement of lipid accumulation during adipogenesis is through the suppression of SIRT3/5, leading to downregulation of the browning effect.

## Discussion

Obesity has been linked to several chronic diseases, cardiometabolic comorbidities, malignancies, and COVID-19 severity (World-Health-Organization, 2021; Chiang et al., 2013; Chiang and Huang, 2014; Chen et al., 2021; Gammone and D'Orazio, 2021). The impaired inner energy regulation and the obesogenic environment lead to the risk of arriving at a pathological state of obesity (Grannell et al., 2021). The majority of excess energy is sequestered as TGs in lipid droplets of WATs. One promising weight-reducing strategy is to increase energy expenditure by activating thermogenic brown and beige adipocytes (Verkerke and Kajimura, 2021). In a pathological environment with high extracellular ATP levels such as obesity, the ATP-activated P2X7 receptor acts as a sensor and arouses accumulating concern in metabolic disorders (Jain and Jacobson, 2021). However, inconsistent directions between P2X7 and adipose tissue accumulation have been reported from experiments using P2X7-deficient or diet-induced obesity mouse models (Sun et al., 2012; Beaucage et al., 2014; Buzzetti et al., 2016; Blasetti Fantauzzi et al., 2017; Giacovazzo et al., 2018). Older male mice lacking P2X7 fed a standard chow diet were found to exhibit adipogenesis and abnormal adipose tissue distribution (Beaucage et al., 2014). Nonetheless, conventional knockout methods would inevitably be confounded by the inactivation of cell types other than adipose tissues. On the other hand, knockdown of CD36 downregulated P2X7 and decreased lipid accumulation in 3T3-L1 cells (Gao et al., 2017). The mRNA levels of *P2X7* in epididymal WATs using a HFD mouse model were significantly increased after six weeks (Sun et al., 2012). Of note, the increased P2X7 expression upon HFD does not necessarily mean activation of the P2X7 receptors and P2X7-mediated signaling pathways (Burnstock, 2006). Therefore, the effect of P2X7 activation on adipogenic and browning genes warrant revisited during adipocyte differentiation.

This study administered the selective P2X7 agonist BzATP and antagonist A438079 in the 3T3-L1 differentiation model to investigate the effects of P2X7 activation and inactivation on two sequential adipogenic factors, PPARγ and C/EBPα. Whether cells were treated at D0/D2 or at stage 2 of adipocyte differentiation, BzATP enhanced the degree of lipid accumulation, intracellular TG formation, and extracellular glycerol release at D4. Although BzATP administration increased mRNA levels of *PPARγ* at D2 and D4, it did not affect protein levels of PPARγ and C/EBPα at D2 or D4. C/EBPα is an essential downstream adipogenic factor of PPARγ (Ghaben and Scherer, 2019). More detailed cascades should be elucidated for the role of P2X7 in these sequential adipogenic factors. In contrast, A438079 treatment at D0/D2 attenuated lipid accumulation, intracellular TG formation, and extracellular glycerol release at D4 without affecting protein and mRNA levels of *PPARγ* and *C/EBPα*. Our results showed the effects of P2X7 on lipid accumulation and metabolism are independent of the expression of adipogenic factors; thus, other determinants such as browning genes should be explored. Given that browning of WAT inhibits lipid accumulation (Bartelt and Heeren, 2014), obesity and HFD feeding, on the contrary, results in a WAT-like morphology in BAT called BAT whitening (Scheja and Heeren, 2019). Obesogenic stimuli and HFD may also increase BAT thermogenic capacity to maintain body weight (García-Ruiz et al., 2015). The feeding-induced mesencephalic astrocyte-derived neurotrophic factor was recently shown to ameliorate diet-induced obesity by promoting adipose browning (Wu et al., 2021). A study detected an increased gene expression of P2X7 not only in WAT but also in whitened BAT of diet-induced obesity and *ob/ob* mice. In contrast, the expression of P2X7 receptors upon 1-day cold exposure was not affected in adipocytes (Tian et al., 2020). Nevertheless, no studies have investigated the association of P2X7 with browning genes during adipocyte differentiation.

PRDM16, PGC-1α, and UCP1 are essential proteins accounting for the browning process, even though UCP1-independent thermogenic pathway has been identified in thermogenic adipocytes (Ikeda and Yamada, 2020). We demonstrated that the mRNA levels of *PRDM16*, *PGC-1α*, and *UCP1* significantly increased at D4. BzATP administration exerted a concentration-dependent inhibition on the enhanced expression of PRDM16, PGC-1α, and UCP-1 at D4, compatible with the observed timing of P2X7 activation on lipid accumulation and metabolism. BzATP treatment at stage 2 of adipocyte differentiation consistently suppressed the mRNA levels of *PRDM16, PGC-1α*, and *UCP1* initiated by stage 2 inducer insulin. Furthermore, BzATP administration during adipogenesis inhibited the protein and mRNA levels of SIRT3/5 at D4. BzATP treatment at stage 2 of adipocyte differentiation also suppressed the mRNA levels of SIRT3 and SIRT5 that were upregulated by insulin. SIRT3 and SIRT5 are located in mitochondria and have been implicated in upregulating the expression of browning genes (Shuai et al., 2019; Gao et al., 2020). SIRT3 may promote brown fat thermogenesis by targeting upstream pathways of UCP1 (Sebaa et al., 2019). SIRT5 was also required for brown adipocyte differentiation (Shuai et al., 2019). Because our data revealed more apparent inhibitory effects on SIRT3/5 mRNA levels exerted by BzATP administration at stage 2 of adipocyte differentiation than at D0/D2, more experiments are needed to understand the detailed mechanisms through which P2X7 downregulates sirtuin gene expression along with the two-stage differentiation period.

In this study, we have provided novel insights into the regulatory roles of P2X7 in lipid accumulation and metabolism during adipocyte differentiation. P2X7 does not directly affect the expression of adipogenic genes during adipocyte differentiation. Instead, P2X7 activation inhibits the expression of browning and SIRT3/5 genes. Taken together, our findings demonstrate that P2X7 enhances lipid accumulation during adipogenesis by suppressing the expression of SIRT3/5 and the browning genes. Further works are necessary to elucidate how P2X7 attenuates the expression of browning and sirtuin genes during adipocyte differentiation. Our findings also warrant verification from primary WAT and BAT of humans and mice. Moreover, the therapeutic potential and window of using selective P2X7 inhibitors in obesity treatment should also be deciphered in the future.

## Data Availability

The original contributions presented in the study are included in the article/Supplementary Material, further inquiries can be directed to the corresponding author.
