# Supplementary material for: P2X7 Activation Enhances Lipid Accumulation During Adipocytes Differentiation Through Suppressing the Expression of Sirtuin-3, Sirtuin-5, and Browning Genes
Source: Front Pharmacol. 2022 Apr 6;13:852858. doi: 10.3389/fphar.2022.852858 (PMC9019299; doi:10.3389/fphar.2022.852858)
Supplement: Supplementary file 1 [file Presentation1.PPTX]

## Slide 1
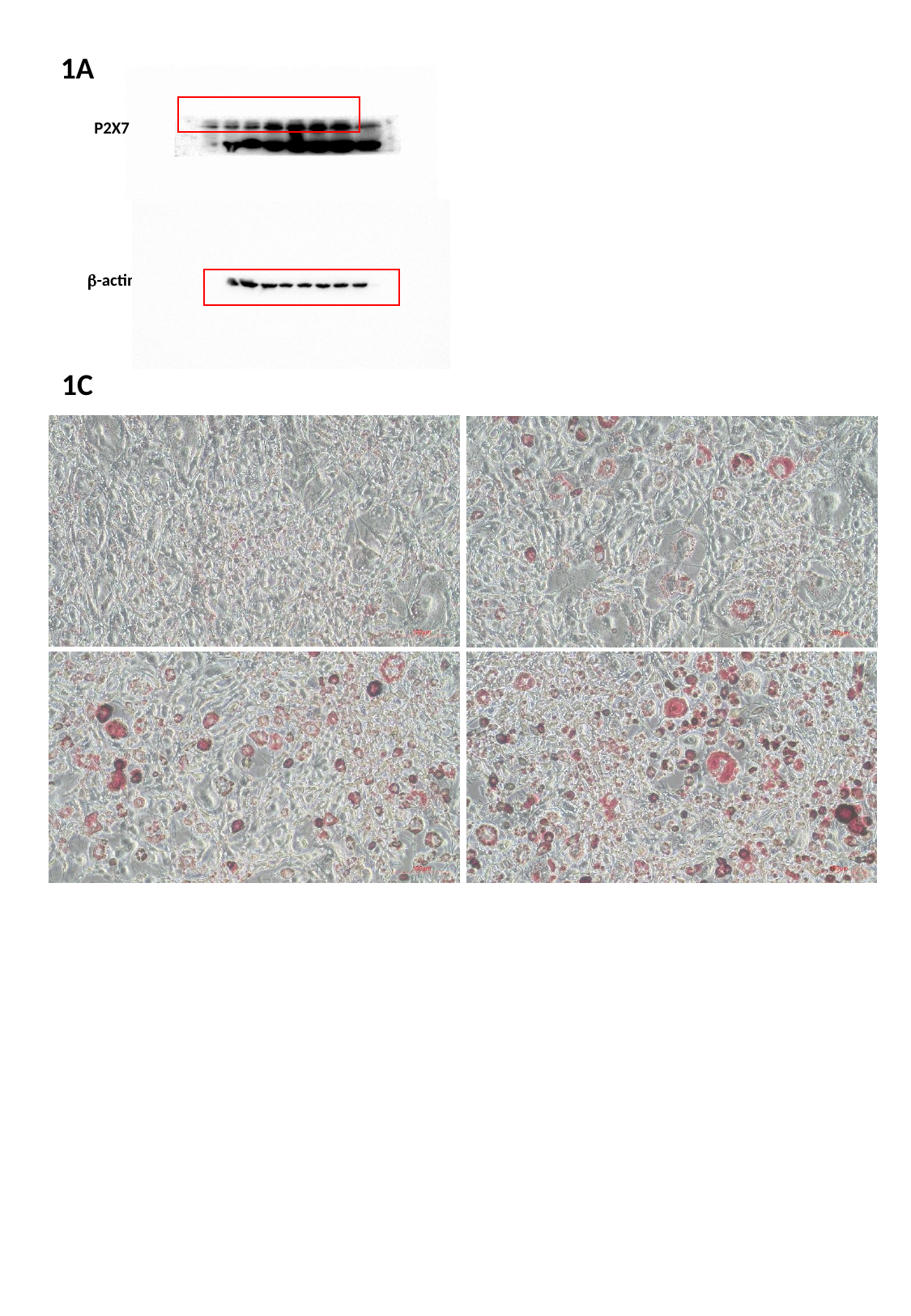

1A
P2X7
b-actin
1C

## Slide 2
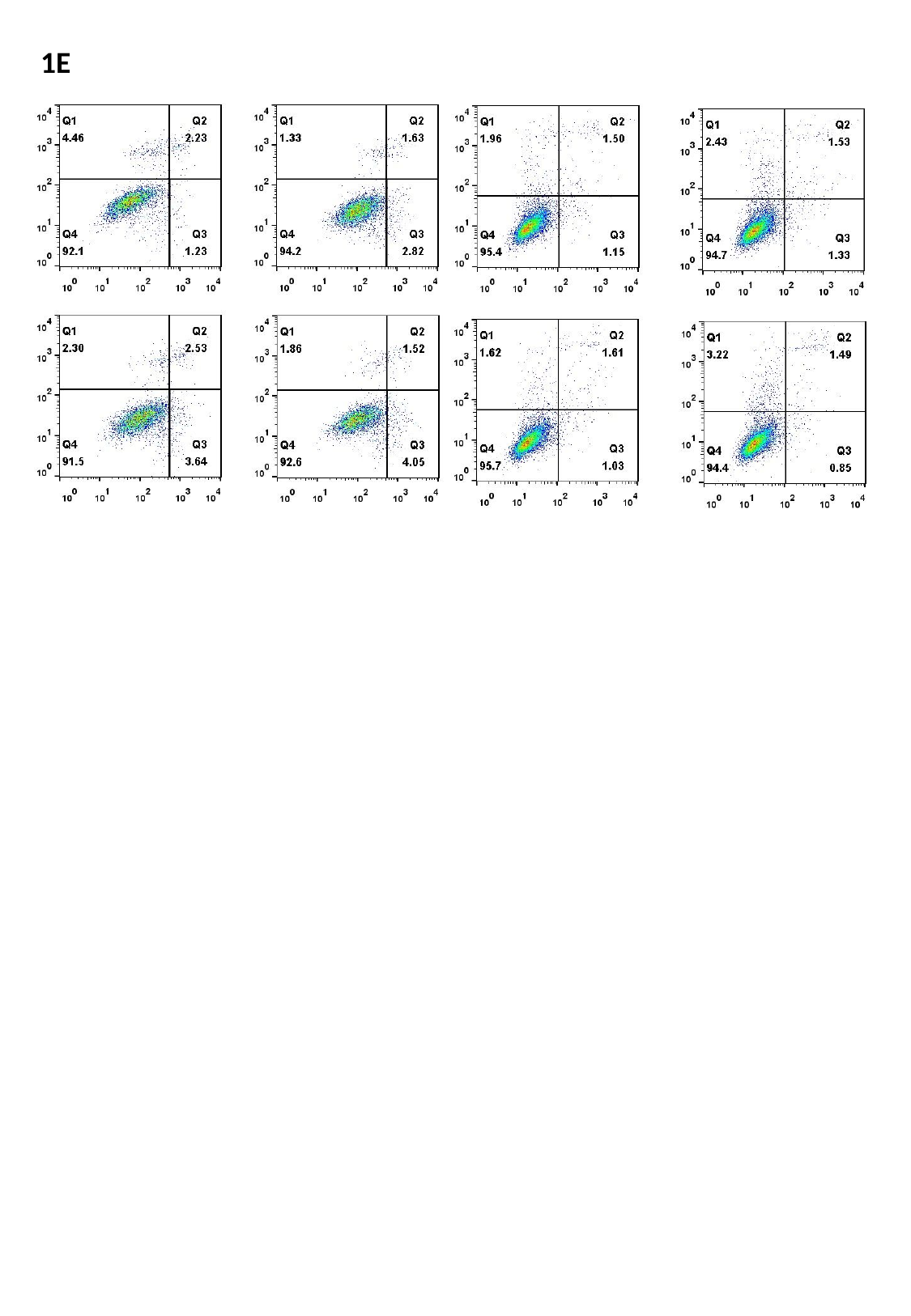

1E

## Slide 3
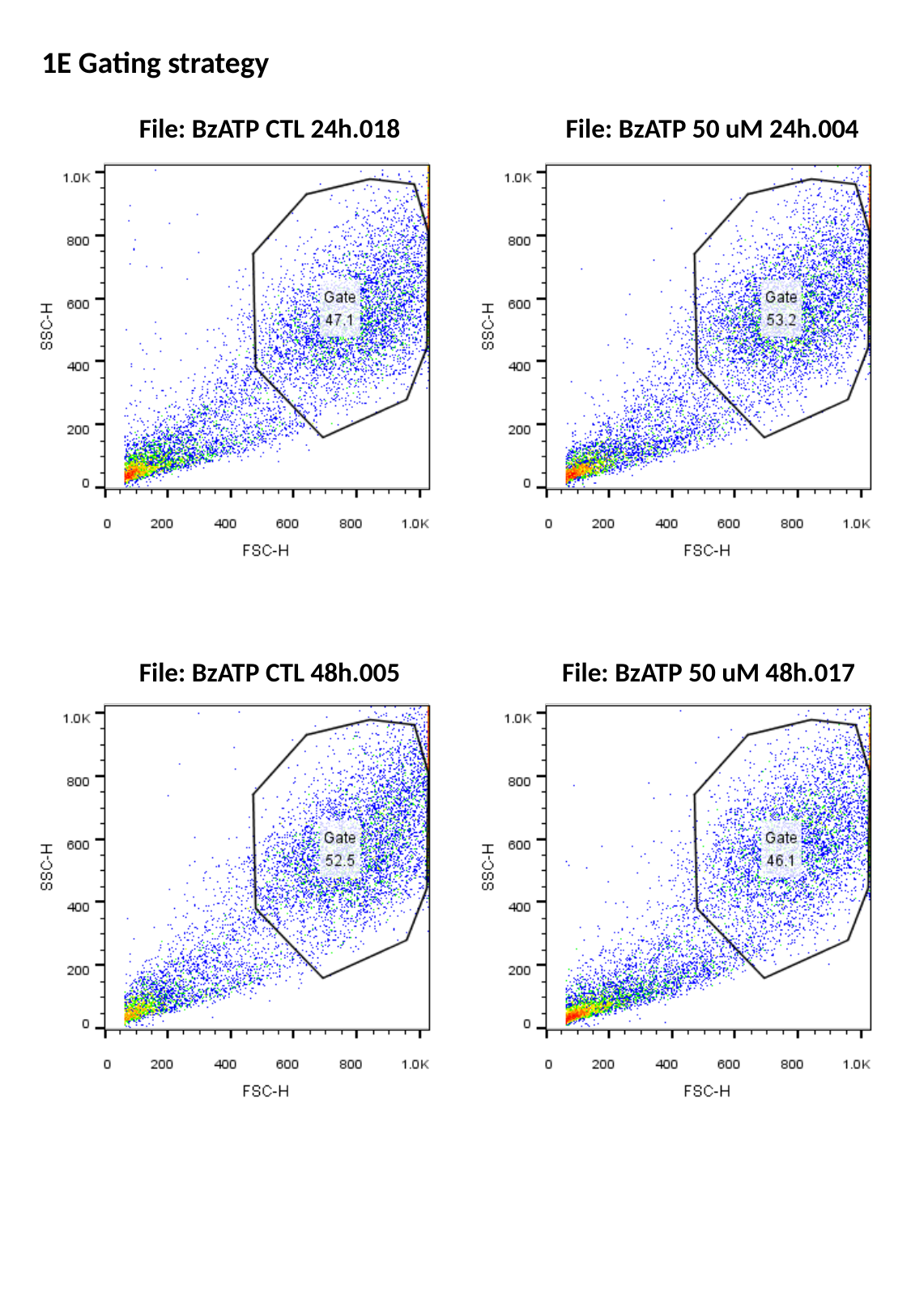

1E Gating strategy
File: BzATP CTL 24h.018
File: BzATP 50 uM 24h.004
File: BzATP CTL 48h.005
File: BzATP 50 uM 48h.017

## Slide 4
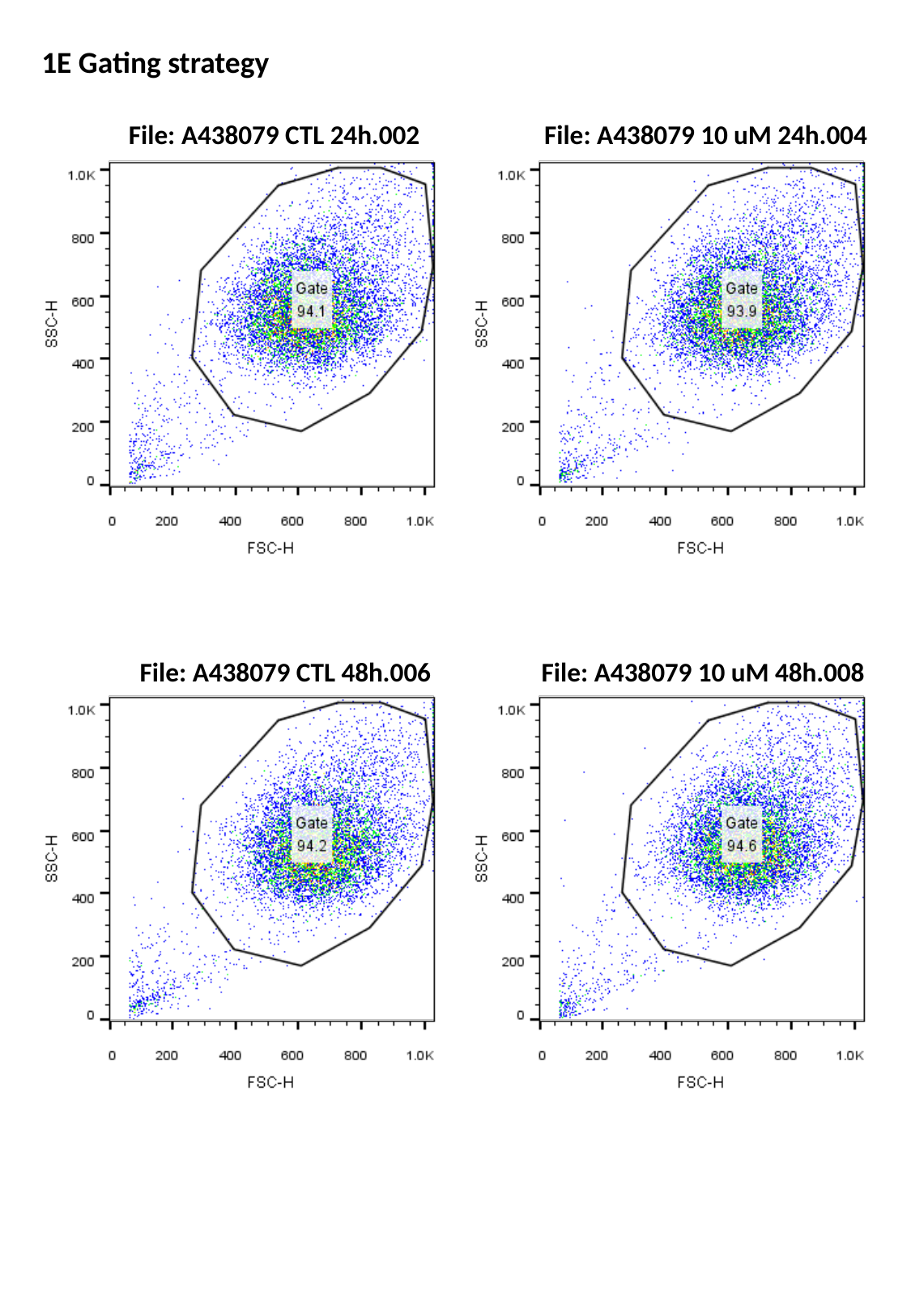

1E Gating strategy
File: A438079 CTL 24h.002
File: A438079 10 uM 24h.004
File: A438079 CTL 48h.006
File: A438079 10 uM 48h.008

## Slide 5
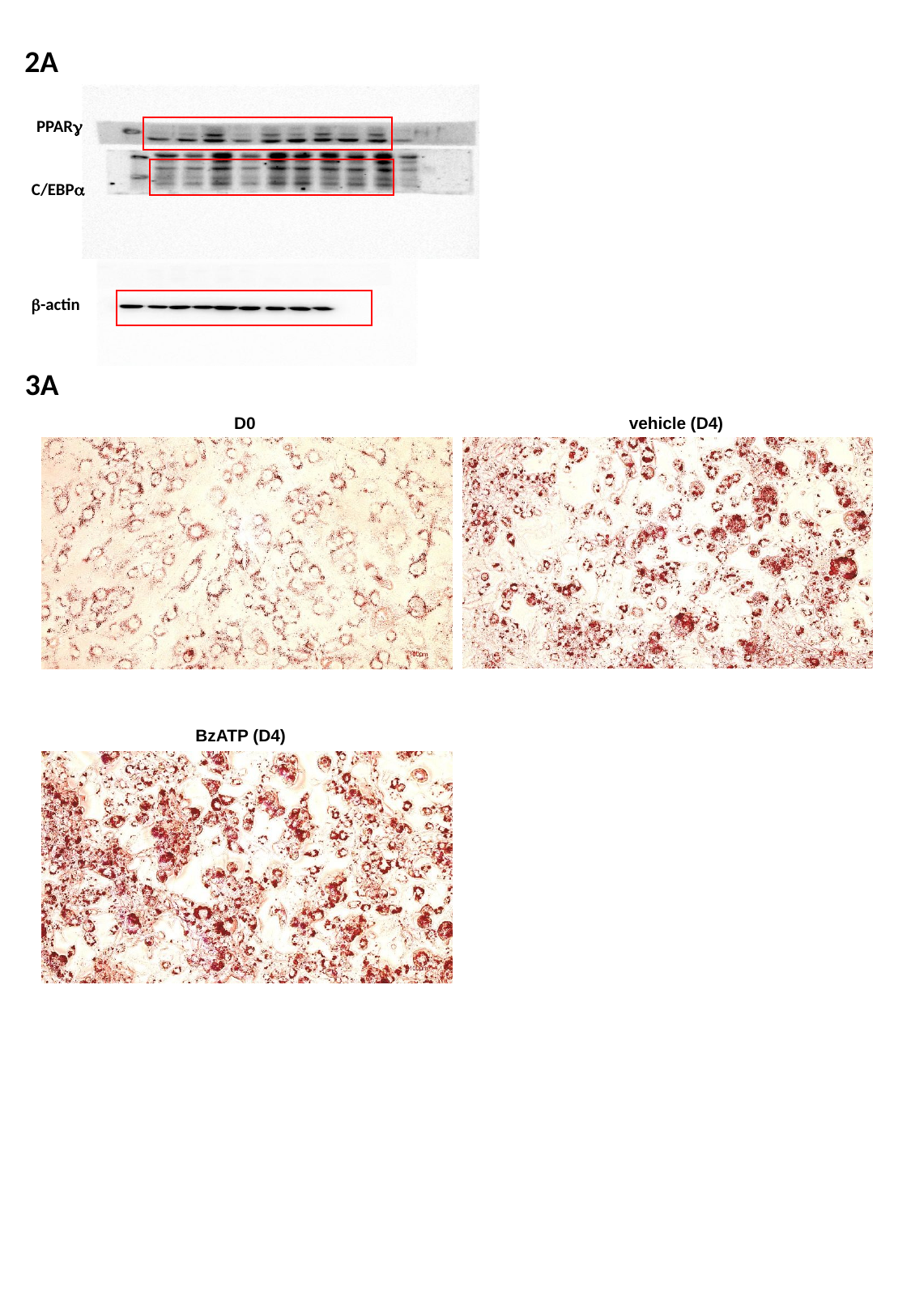

2A
PPARg
C/EBPa
b-actin
3A
D0
vehicle (D4)
BzATP (D4)

## Slide 6
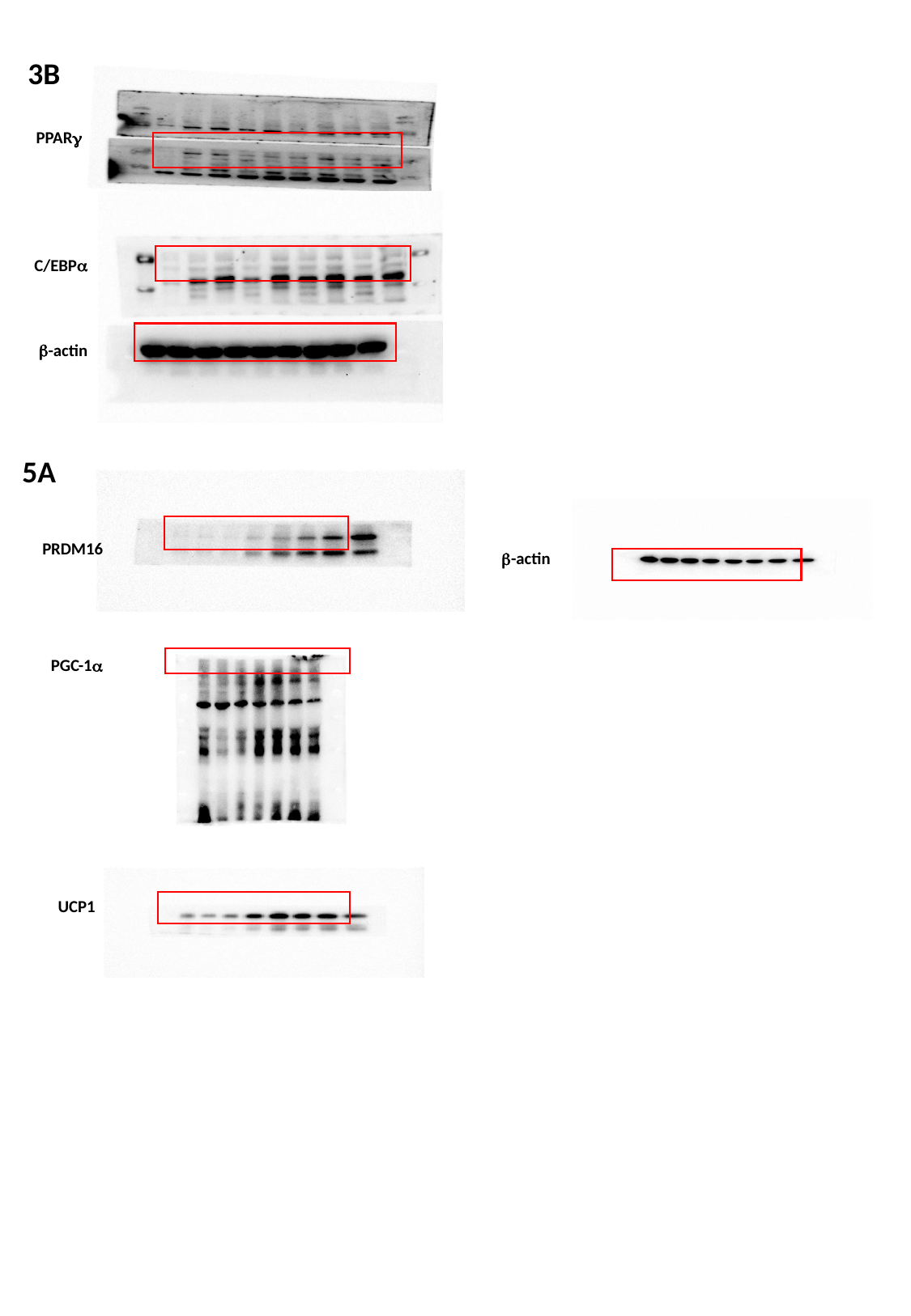

3B
PPARg
C/EBPa
b-actin
5A
PRDM16
b-actin
PGC-1a
UCP1

## Slide 7
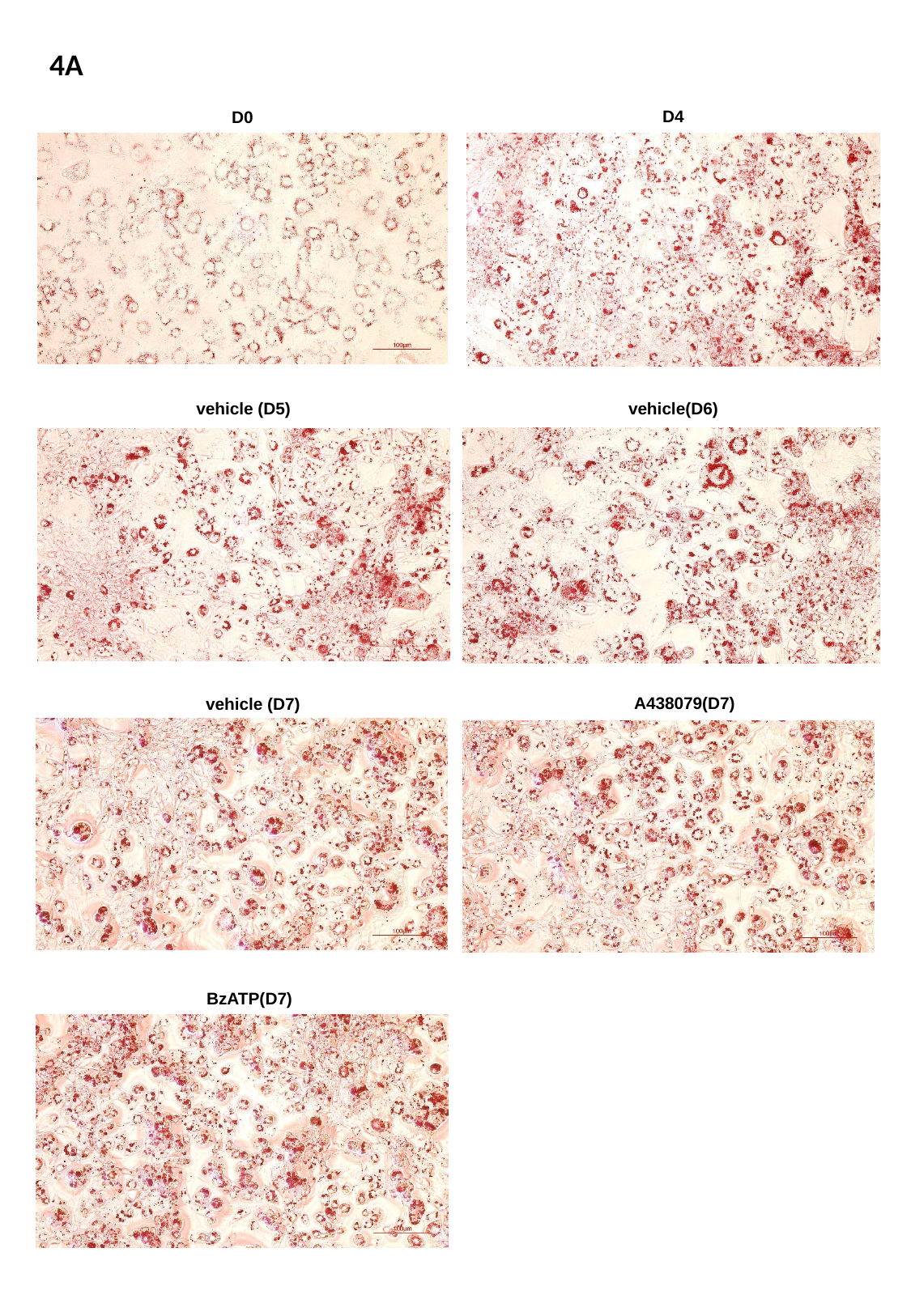

4A
D4
D0
vehicle(D6)
vehicle (D5)
A438079(D7)
vehicle (D7)
BzATP(D7)

## Slide 8
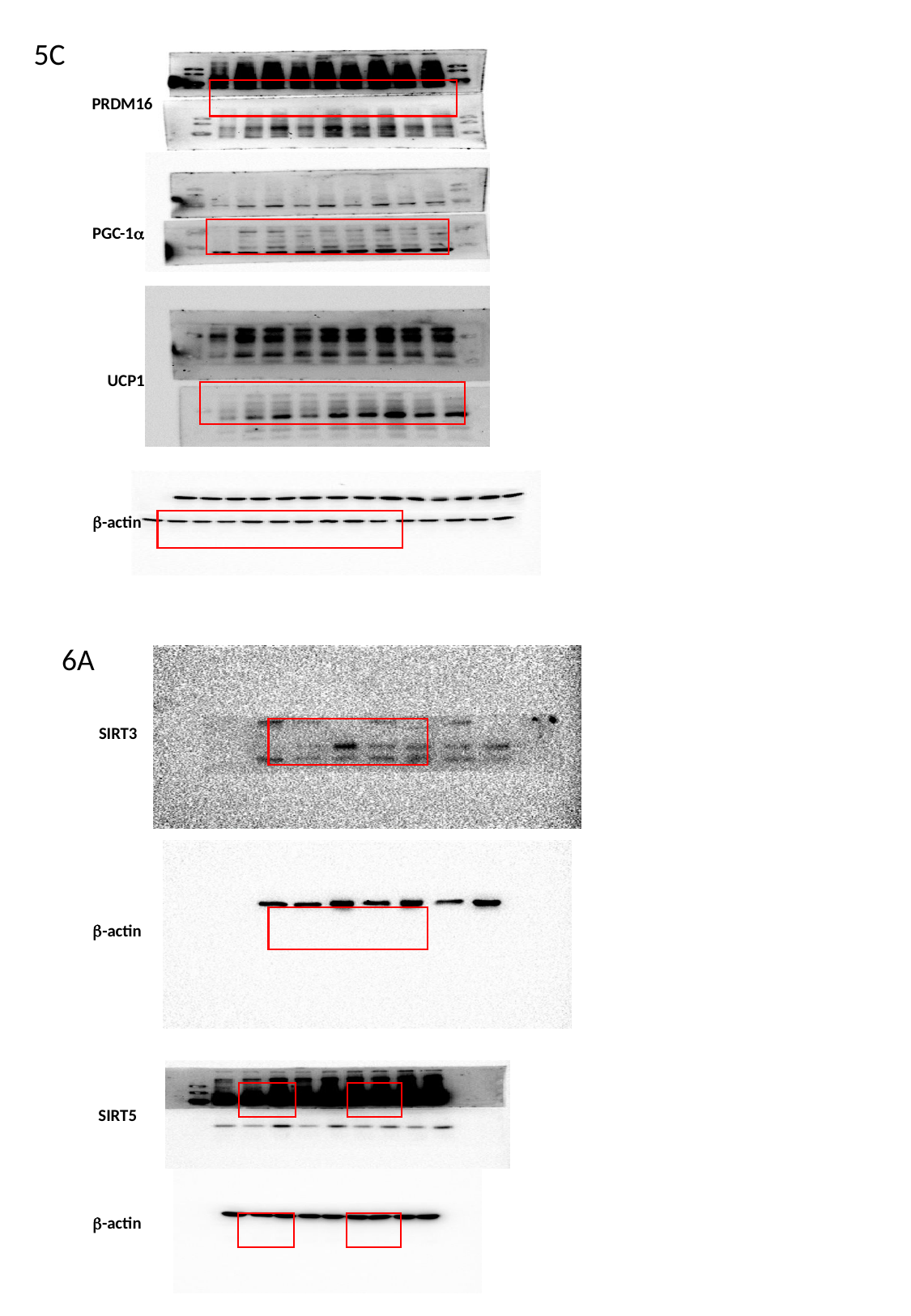

5C
PRDM16
PGC-1a
UCP1
b-actin
6A
SIRT3
b-actin
SIRT5
b-actin
