# Supplementary material for: P2X7 Activation Enhances Lipid Accumulation During Adipocytes Differentiation Through Suppressing the Expression of Sirtuin-3, Sirtuin-5, and Browning Genes
Source: Front Pharmacol. 2022 Apr 6;13:852858. doi: 10.3389/fphar.2022.852858 (PMC9019299; doi:10.3389/fphar.2022.852858)

**Raw source data (.fcs) for all flow cytometry experiments and download links**

File: BzATP CTL 24h.018

<https://www.space.ntu.edu.tw/navigate/s/58D9812B32EB452BB17E75097D1FF7D7QQY>

File: BzATP CTL 48h.005

<https://www.space.ntu.edu.tw/navigate/s/82FBF31737C343C9B78CF310968E8B74QQY>

File: BzATP 50 uM 24h.004

<https://www.space.ntu.edu.tw/navigate/s/0634D37F2F2C4347A06B2C8447367DF7QQY>

File: BzATP 50 uM 48h.017

<https://www.space.ntu.edu.tw/navigate/s/D74B8D0B548740C4817005A5D97ED229QQY>

A438079 10 uM 24h.004

<https://www.space.ntu.edu.tw/navigate/s/538EE46FCA2644698DEC8E50956718EBQQY>

A438079 10 uM 48h.008

<https://www.space.ntu.edu.tw/navigate/s/BF050ABBCADF4CB9913DAB9E97D66BBEQQY>

A438079 CTL 24h.002

<https://www.space.ntu.edu.tw/navigate/s/554FC394F13A4D39B16D52EDC2F86B58QQY>

A438079 CTL 48h.006

<https://www.space.ntu.edu.tw/navigate/s/724C1EAA62C0472D92948228C2B75711QQY>

Please note:

We could NOT set the sharing scope for any download links of our raw source data at https://www.jianguoyun.com/ to “Anyone” because only phone numbers in the Chinese Mainland are available for getting share links.
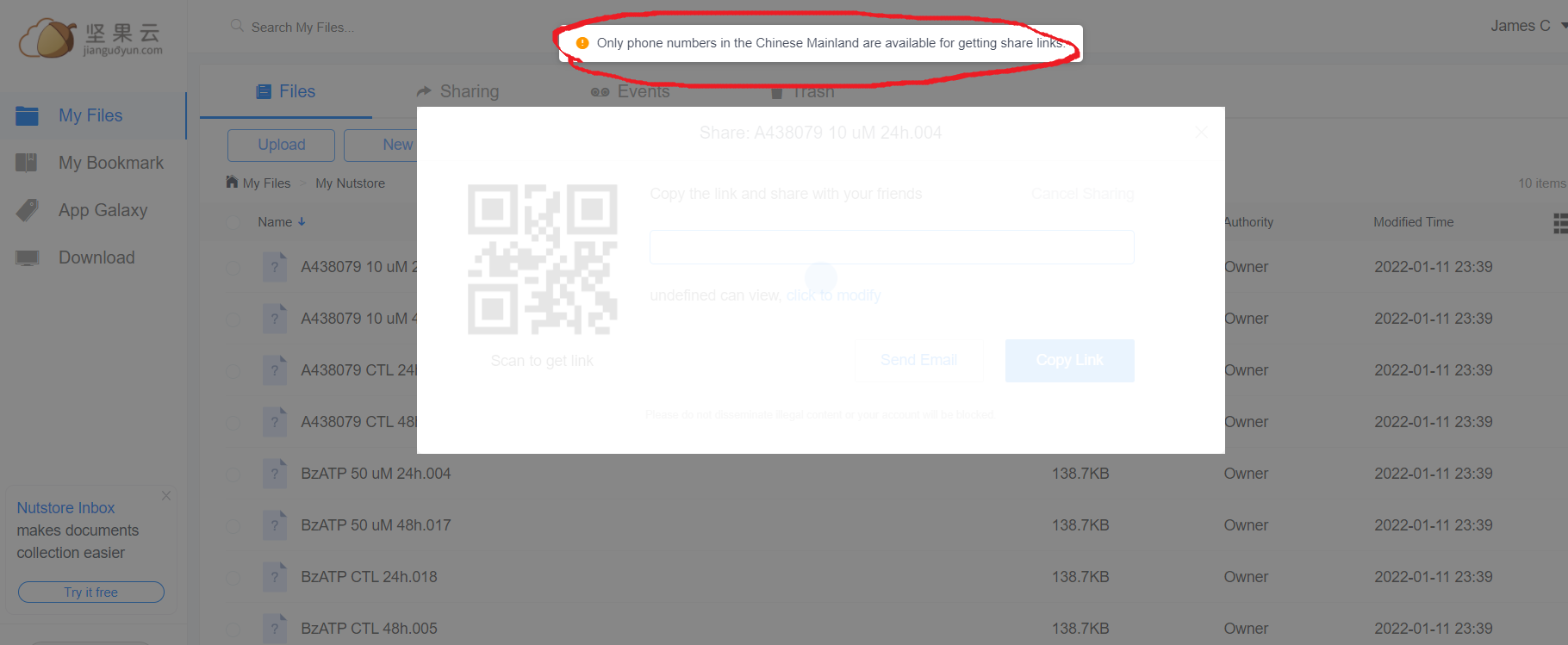

Supplement: Supplementary file 2 [file DataSheet2.DOCX]
